# Identifying Patient Sentiment in Atopic Dermatitis Treatment: Large Language Model Approach

**DOI:** 10.2196/78054

**Published:** 2026-01-02

**Authors:** Jack Alexander Cummins, JiaDe Yu

**Affiliations:** 1 Princeton University Princeton, NJ United States; 2 Department of Dermatology Massachusetts General Hospital Boston, MA United States

**Keywords:** atopic dermatitis, eczema, large language model, social media, dupilumab, upadacitinib, abrocitinib, tralokinumab

## Abstract

This study demonstrates that GPT-4o outperforms traditional natural language processing methods in accurately analyzing patient sentiment toward atopic dermatitis treatments on Reddit, enabling more nuanced and reliable extraction of real-world patient perspectives from large-scale social media data.

## Introduction

Atopic dermatitis (AD) treatment has broadened since 2017, with several new, targeted, highly efficacious systemic therapies. Patients’ personal experiences with these novel therapies are largely unknown and unreported. Reddit is a rich source of patient perspectives on dermatology [1]. Previous studies have used traditional natural language processing (NLP) methods to extract meaningful information from unstructured social media data [2], but more relevant findings could potentially be extracted by applying large language models (LLMs) to such language data, including large-scale Reddit datasets, because tailored prompts can extract more specific and nuanced insights.

## Methods

### Overview

We used the Pushshift Reddit dataset to access all Reddit comments (n=8,543,388) posted prior to January 1, 2024, on various dermatology-related subreddits (Multimedia Appendix 1, Table S1). We analyzed all comments containing the generic or brand name of four AD therapies as of January 1, 2024: dupilumab, upadacitinib, abrocitinib, and tralokinumab. This resulted in 27,272 comments. Our novel approach applied OpenAI’s developer application programming interface (API) to access GPT-4o [3], OpenAI’s cutting-edge LLM, for sentiment analysis to determine whether comments indicated positive, neutral, or negative impressions of the medications. The GPT-4o API was configured with a temperature setting of 0.0 to ensure consistent and deterministic responses across all analyses, eliminating variability in sentiment classification. The complete prompt used for the GPT-4o sentiment analysis is provided in Multimedia Appendix 2.

### Model Comparison

We compared 3 automated tools against manual sentiment analysis to identify the most accurate tool. Two dermatologists (JY and Hadley Johnson, MD) independently reviewed 100 randomly selected comments (25 comments for each medication) and reached concordance for 84 of 100 sentiments (κ=0.75, concordance=0.84). The dermatologists classified the comments as positive, neutral, or negative based on their overall expressed sentiment toward the medication without predefined annotation guidelines. Disagreements typically occurred for comments containing ambiguous treatment responses, mixed sentiments about medication efficacy, or informational discussions rather than clear personal sentiment expressions. The 84 comments were used as our reference standard for testing GPT-4o and two traditional NLP sentiment analysis methods: Valence Aware Dictionary and Sentiment Reasoner (VADER) [4], a lexicon-based tool specifically attuned to social media text, and distilbert-base-uncased-finetuned-sst-2-english (DistilBERT), a model pre-finetuned on the Stanford Sentiment Treebank for Sentiment Analysis [5]. The DistilBERT model was accessed through the Hugging Face Transformers library using its high-level pipeline interface. VADER and DistilBERT were used with default parameters. These models were selected as commonly used general-purpose sentiment analysis tools.

### LLM Application

We applied GPT-4o to evaluate the sentiment of the posts as positive, neutral, or negative. This resulted in 28,889 total analyses, as some comments listed more than one medication. CIs were calculated for the proportion of positive comments and the proportion of negative comments using a binomial proportion CI.

Based on the high κ statistic (κ=0.73) of the LLM for predicting sentiment, we used GPT-4o to analyze sentiment in the full set of 27,272 comments mentioning the medications (Multimedia Appendix 3, Figure S1).

### Ethical Considerations

This study analyzed publicly available, deidentified Reddit comments and does not constitute human subjects research as it involved secondary analysis of existing public data that cannot be linked to identifiable individuals.

## Results

**Figure 1:**
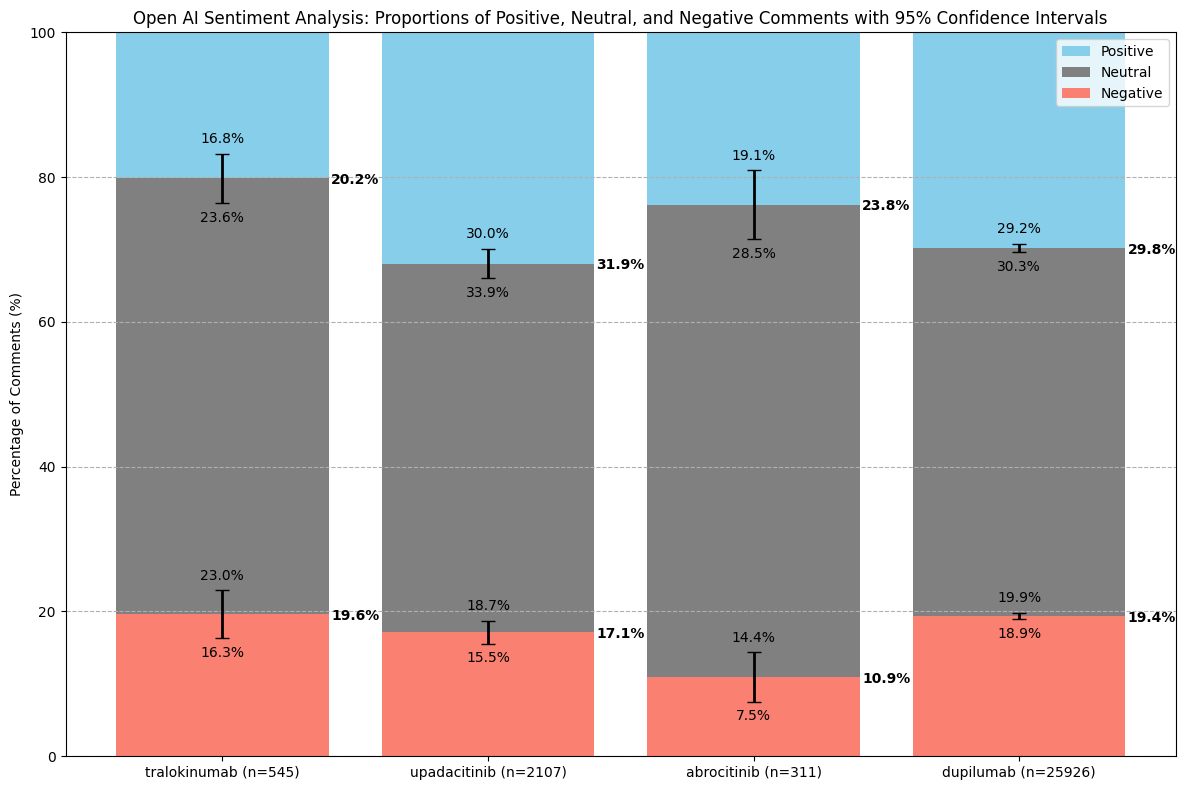
Bar chart showing the proportion of positive, neutral, and negative comments for each therapy, as identified by GPT-4o. CIs were calculated for the positive and negative bars using binomial proportion CIs.

GPT-4o demonstrated superior performance across standard classification metrics (Table 1), with precision of 0.87, recall of 0.82, *F*_1_-score of 0.82 (support-weighted across classes), and accuracy of 0.82, compared to VADER (precision: 0.42; recall: 0.38; *F*_1_-score: 0.37; accuracy: 0.38) and DistilBERT (precision: 0.58; recall: 0.56; *F*_1_-score: 0.56; accuracy: 0.56). The medications with the highest proportion of comments in the full dataset tagged as positive by GPT-4o (Figure 1) were upadacitinib (673/2107, 31.9%) and dupilumab (7724/25,926, 29.8%). Abrocitinib had a smaller percentage of negative sentiments than other medications (Figure 1). Dupilumab and upadacitinib had a high percentage of positive sentiment, which may suggest that many individuals are satisfied with the efficacy and safety of these medications.

Examples from the dataset illustrate the range of patient experiences. Positive comments expressed enthusiasm about treatment options, such as one patient’s response to abrocitinib approval: “Just saw it was just approved! Calling my dermo on Monday!” Negative sentiment reflected disappointment with treatment outcomes, as seen in one dupilumab user’s comment: “I thought Dupixent would provide relief too, but I’m still itchy and inflamed.” Neutral comments predominantly involved informational exchanges, such as questions about accessing treatment and questions about clinical trial participation.

**Table 1:** In the initial subset of 84 manually evaluated comments, the large language model–based approach showed a remarkably stronger correlation with human judgment (κ=0.73) than distilbert-base-uncased-finetuned-sst-2-english (DistilBERT; κ=0.33) or Valence Aware Dictionary and Sentiment Reasoner (VADER; κ=0.06).

Model agreement with dermatologist judgment	GPT-4o	VADER	DistilBERT
Positive comments (n=26), n (%)	24 (92)	16 (62)	15 (58)
Neutral comments (n=41), n (%)	28 (68)	13 (32)	21 (51)
Negative comments (n=17), n (%)	17 (100)	3 (18)	11 (65)

## Discussion

GPT-4o showed substantially better agreement with clinician judgment than traditional NLP methods when classifying patient sentiment in Reddit comments about AD treatments. The superior performance across all metrics establishes the feasibility of applying LLMs to extract patient perspectives from unstructured social media data. However, it is important to note that we excluded comments when the annotators disagreed to ensure a clear reference standard. This approach potentially biased the validation set toward straightforward sentiment examples and likely inflated the reported performance metrics, as the models were not tested on ambiguous, mixed-emotion, or context-dependent language, which is common in real patient discussions. Simple task-specific prompts applied to LLMs may yield more detailed insights than traditional NLP methods. LLMs offer advantages through basic zero-shot prompts that can be adapted for specific tasks like drug-specific sentiment analysis in medical discussions, whereas general-purpose sentiment analysis tools may struggle with medical terminology and context without additional training or customization. However, LLM-based approaches also have limitations, including computational costs and the need for careful prompt design to ensure reliable results. The predominance of neutral sentiment across all medications shows that many comments serve informational or question-asking purposes rather than expressing clear sentiment. Clinically, the observed higher positive sentiment for upadacitinib and dupilumab in our dataset may reflect patient satisfaction with these treatments, while the lower negative sentiment observed with abrocitinib could indicate fewer patient-reported concerns. Similar approaches could illuminate real-world perspectives on treatments for psoriasis and other chronic skin conditions where multiple therapeutic options exist. While our findings provide initial insights for AD treatment discussions, they also suggest potential broader applications for analyzing patient perspectives across medical fields. Future studies should expand beyond simple sentiment categorization to capture more nuanced patient experiences, including mixed sentiments, specific concerns about side effects, cost considerations, and conditional satisfaction with treatment. Additionally, future research should explore temporal trends in sentiment as medications gain market share and correlate social media sentiment with real-world evidence databases and patient registries.

## Appendix

Multimedia Appendix 1 List of dermatology subreddits used in the study and the total number of comments in each subreddit prior to January 1, 2024.

Multimedia Appendix 2 Prompt used by GPT-4o to generate results.

Multimedia Appendix 3 Flow chart displaying the system workflow for the study.

## Abbreviations

AD: atopic dermatitis
API: application programming interface
DistilBERT: distilbert-base-uncased-finetuned-sst-2-english
LLM: large language model
NLP: natural language processing
VADER: Valence Aware Dictionary and Sentiment Reasoner
