# Supplementary material for: Identifying Patient Sentiment in Atopic Dermatitis Treatment: Large Language Model Approach
Source: JMIR Form Res. 2026 Jan 2;10:e78054. doi: 10.2196/78054 (PMC12811741; doi:10.2196/78054)
Supplement: Multimedia Appendix 1 [file formative_v10i1e78054_app1.docx]

| **Subreddit** | **Number of Comments** |
| --- | --- |
| r/SkincareAddiction | 6,190,706 |
| r/Skincare_Addiction | 538,357 |
| r/SkincareAddicts | 532,644 |
| r/eczema | 466,769 |
| r/DermatologyQuestions | 336,651 |
| r/Dermatology | 227,721 |
| r/SkincareAddictionUK | 182,073 |
| r/TS_Withdrawal | 48,836 |
| r/eczeMABs | 19,631 |

*Table S1. List of dermatology subreddits used in the study and the total number of comments in each subreddit prior to January 1, 2024*
