# Supplementary material for: Identifying Patient Sentiment in Atopic Dermatitis Treatment: Large Language Model Approach
Source: JMIR Form Res. 2026 Jan 2;10:e78054. doi: 10.2196/78054 (PMC12811741; doi:10.2196/78054)
Supplement: Multimedia Appendix 2 [file formative_v10i1e78054_app2.docx]

**Prompt**

Classify the sentiment of this comment about "{drug}" (generic: "{generic}") as one of the following:

- Positive

- Neutral

- Negative

Do not include explanations or any additional text. Just respond with one of the three labels.

Comment: "{comment}"
