# Supplementary figures and images for: Identifying Patient Sentiment in Atopic Dermatitis Treatment: Large Language Model Approach
Source: JMIR Form Res. 2026 Jan 2;10:e78054. doi: 10.2196/78054 (PMC12811741; doi:10.2196/78054)

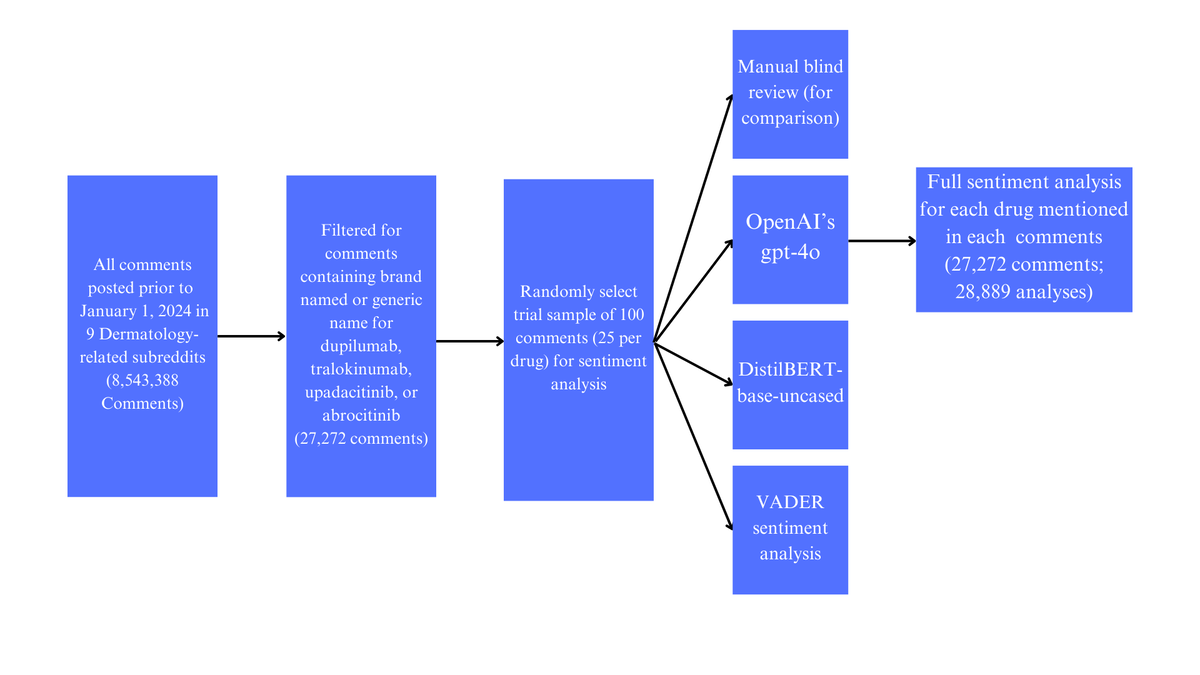

Supplement: Multimedia Appendix 3 [file formative_v10i1e78054_app3.png]
